# The Short-Term Performances of Two Independent Gas Modulated Refractometers for Pressure Assessments

**DOI:** 10.3390/s21186272

**Published:** 2021-09-18

**Authors:** Clayton Forssén, Isak Silander, Johan Zakrisson, Ove Axner, Martin Zelan

**Affiliations:** 1 Department of Physics, Umeå University, SE-901 87 Umeå, Sweden; clayton.forssen@umu.se (C.F.); isak.silander@umu.se (I.S.); johan.zakrisson@umu.se (J.Z.); ove.axner@umu.se (O.A.); 2 Measurement Science and Technology, RISE Research Institutes of Sweden, SE-501 15 Borås, Sweden

**Keywords:** refractometry, pressure, short-term performance, Fabry–Perot cavity, gas modulation, modulation techniques, metrology

## Abstract

Refractometry is a powerful technique for pressure assessments that, due to the recent redefinition of the SI system, also offers a new route to realizing the SI unit of pressure, the Pascal. Gas modulation refractometry (GAMOR) is a methodology that has demonstrated an outstanding ability to mitigate the influences of drifts and fluctuations, leading to long-term precision in the $10^{-7}$ region. However, its short-term performance, which is of importance for a variety of applications, has not yet been scrutinized. To assess this, we investigated the short-term performance (in terms of precision) of two similar, but independent, dual Fabry–Perot cavity refractometers utilizing the GAMOR methodology. Both systems assessed the same pressure produced by a dead weight piston gauge. That way, their short-term responses were assessed without being compromised by any pressure fluctuations produced by the piston gauge or the gas delivery system. We found that the two refractometer systems have a significantly higher degree of concordance (in the $10^{-8}$ range at 1 s) than what either of them has with the piston gauge. This shows that the refractometry systems under scrutiny are capable of assessing rapidly varying pressures (with bandwidths up to 2 Hz) with precision in the $10^{-8}$ range.

## 1. Introduction

Refractometry is a powerful technique for assessing gas pressure. It is based upon measuring, by optical means, the change in refractive index in a measurement compartment as gas is let into it. From the change in refractive index, under the condition that the molar polarizability (and higher order refractive virial coefficients) of the gas is known, the change in gas density can be calculated. From this, provided that the gas temperature is known, the pressure can be assessed by utilizing an equation of state. Moreover, since the Boltzmann constant was given a fixed value (i.e., without uncertainty) in the 2019 revision of the SI system of units [1], refractometry also offers a new and independent route to realizing the SI unit of pressure, i.e., the Pascal [2]. These exciting prospects have spurred a significant increase of interest within the field of refractometry. Work to explore and utilize the potential of optical methods for assessing the molar density and pressure of gas presently takes place at several national metrology institutes and universities [2,3,4,5,6,7,8,9].

Besides being a potential primary method for measuring the Pascal, the technology also has several other highly interesting properties and advantages. As optical measurements do not utilize any mechanical actuators, the highest pressures that can be measured tend to be limited by the gas handling system used. The lowest pressure shifts that can be resolved are in turn limited by the laser locking. In practical terms, the dynamic range can be as high as eight orders of magnitude, typically covering the range from 1 mPa to 100 kPa. As optical measurements are performed by measuring changes in frequency, which can be measured swiftly and continuously with high accuracy, these systems can also be designed to give measurements of pressure with high time resolution. In practice, while the long-term performance is given by the stability of the cavity spacer, the time resolution and short-term performance are given by the acquisition rate and stability of the frequency counter. This combination of an extraordinary large dynamic range and a fast response facilitate accurate assessments of large pressure shifts with short settling times. Such measurements can be used to characterize pressure sensors, resolve the differences between sensor responses and actual pressure changes, study rapidly changing pressures, and investigate processes giving rise to such.

The best performing refractometers are based on Fabry–Perot (FP) cavities, where a laser is used to probe the frequency of a longitudinal cavity mode [5,6,7,8,9,10,11,12,13,14,15]. By measuring the change in frequency between an empty (evacuated) and a gas-filled cavity, the refractivity can be assessed, from which the molar density and the pressure can be calculated. However, since such measurements cannot distinguish changes in refractivity of a gas from drifts in the physical length of the cavity, the most sophisticated FP refractometers utilize a dual FP cavity (DFPC) design in which one cavity acts as the measurement cavity and the other as a reference. This eliminates common-mode drifts in the cavity spacer caused by aging, temperature drifts, mechanical stress, etc. However, since the two cavities in a DFPC can drift dissimilarly, extraordinarily stable conditions are still required to achieve optimal performance. For a 15 cm long cavity, a drift in length of 1 pm gives rise to a shift in the assessed pressure of nitrogen of 2.5 mPa. As a means to remedy this, we developed a measurement methodology denoted gas modulation refractometry (GAMOR) [6,16,17,18,19].

The GAMOR methodology is based on repeated measurements performed on a relatively short timescale (typically using gas filling and evacuating cycles of 100 s) combined with an interpolation procedure in which the empty measurement cavity response is taken as the interpolated value of two such measurements—one taken just before and one directly after the filled measurement cavity measurement. That way, the influences of both long-term drifts and various types of fluctuations can be strongly suppressed [6,20,21]. Furthermore, the influences of leaks and outgassing in the reference cavity can automatically be corrected for.

In order to perform high accuracy refractometry based on the GAMOR principle, care needs to be taken regarding the construction of the refractometers. To enable repeated filling and emptying of gas on relatively short time scales without introducing excessive amounts of PV work, cavities with small volumes have been implemented (<5 cm${}^{3}$) [17,22]. The cavity spacers in these works were made from Invar which has both higher thermal conductivity and a larger volumetric heat capacity than commonly used glass materials (Zerodur and ULE glass).

This does not only eliminate any possible heat islands in the system; it also facilitates the assessment of gas temperature, which is performed by measurement of the temperature of the cavity spacer by the use of temperature probes placed in drilled holes in, or in direct contact with, the spacer. The use of Invar also eliminates effects of gas permeation which have been reported for ULE glass [5,23]. Furthermore, to allow for fully automatic operation with sturdy laser locking and automated mode jumps, systems based on rugged narrow-banded fiber lasers working in the near IR (NIR) communication region (around 1.55 µm) have been used [6].

This has lead to instrumentation that is capable of providing measurements with precision in the sub-ppm (sub-parts-per-million or sub-$10^{-6}$) range [6,16,17,18]. By then also using well-calibrated temperature sensors and accurately assessed molecular parameters (molar polarizabilties and virial coefficients), the systems can demonstrate good accuracy. Such a system, denoted the stationary optical pascal (SOP), was recently characterized in terms of its ability to realize the Pascal [19]. It was found that its uncertainty was [(10 mPa)${}^{2}$ + (10 × 10${}^{-6}{P)}^{2}$]${}^{1/2}$, mainly limited by the uncertainty in the molar polarizability of nitrogen (8 ppm) [19].

To assess the ability to realize a transportable refractometer, a similar system, denoted the transportable optical pascal (TOP), was recently developed and characterized. It was found that its uncertainty was [(16 mPa)${}^{2}$ + (28 × 10${}^{-6}P{{)}^{2}]}^{1/2}$, mainly limited by the uncertainty of the temperature probes used for assessment of the temperature (26 ppm) [19].

As was alluded to above, to make viable assessments of large pressure shifts with short settling times, which is needed for a number of applications, it is of importance that the system has a fast response. Although several types of refractometers have been scrutinized over the years [3,4,5,6,7,8,10,11,12,13,14,15,16,17,18,19,24,25,26], virtually none of them has yet been assessed with respect to its short-term behavior. Access to two GAMOR-based refractometer systems allows for scrutiny of the short-term behavior of GAMOR-based refractometry in more detail. By comparing two fully independent GAMOR-based refractometer systems (the aforementioned SOP and TOP systems) connected to the same gas system, whose pressure was set by a dead weight piston gauge (DWPG), their short-term performances could be scrutinized in some detail. As the refractometers were completely independent, it could be concluded that deviations that are common to both systems are not inherent to one or the other refractometer, but rather the DWPG and/or the gas handling system. Thereby, we could ascertain the precision of the refractometers without any influence from the DWPG or gas handling system. Indeed, we assessed the short-term performances of two independent gas modulated refractometers regarding their ability to assess pressure. It was found that the refractometers can provide short-term precision on the 1 s time scale of $3\times 10^{-8}$, which is one order of magnitude better than the corresponding stability of the pressure provided by the DWPG. This opens up a number of novel applications for refractometry.

Although the SOP refractometer previously has been well described [17,25], the TOP system has not. This system, including its construction and various components, is therefore described in some detail here. In addition, the theoretical model used for the evaluation of the data gathered is provided.

## 2. Theory

### 2.1. Refractivity

As has previously been outlined [19], each DFPC refractometer addresses the empty cavity mode $q_{01}$ or $q_{02}$ with light of frequency $\nu_{01}$ or $\nu_{02}$, respectively. The beat frequency between the two lasers, *f*, which is the measured entity, is given by the difference between the two laser frequencies, defined as $|\nu_{1}-\nu_{2}|$. Since the lasers have a limited tuning range, automatic mode jumps will take place when the change in pressure becomes large. This implies that *f* is a non-monotonic (i.e., a wrapped) function. It is therefore convenient to define an unwrapped beat frequency as

(1) $$f_{UW}=\pm f-\left(\frac{\Delta q_{1}}{q_{01}}\nu_{01}-\frac{\Delta q_{2}}{q_{02}}\nu_{02}\right),$$

where $\Delta q_{1}$ and $\Delta q_{2}$, counted from $q_{01}$ and $q_{02}$, are the mode jumps and where the ± sign refers to the cases when $\nu_{1}>\nu_{2}$ and $\nu_{1}<\nu_{2}$.

The refractivity can then be expressed as a function of the shift of the unwrapped beat frequency when gas is let out of (or into) the measurement cavity, $\Delta f_{UW}$. As has been shown recently [19], while denoting the measurement cavity as *m*, the refractivity can be expressed as a function of the unwrapped beat frequency when GAMOR is used as

(2) $$n-1=\frac{|\Delta f_{UW}|/v_{0m}}{1-|\Delta f_{UW}|/v_{0m}+\Delta q_{m}/q_{0m}+\varepsilon_{m}},$$

where $\varepsilon_{m}$ is a deformation parameter comprising the refractivity-normalized relative difference in lengths of the two cavities due to pressurization, given by $\left[{\left(\delta L/L_{0}\right)}_{m}-{\left(\delta L/L_{0}\right)}_{r}\right]/\left(n-1\right)$, where ${\left(\delta L/L_{0}\right)}_{m}$ and ${\left(\delta L/L_{0}\right)}_{r}$ are the relative changes in length of the measurement and reference cavities when the measurement cavity is pressurized [6,16,27]. It is worth noting that $\varepsilon_{m}$ can be assessed with high accuracy by a methodology developed by Zakrisson et al. [26].

In Equation (2) the influences of the mirror dispersion and the finite penetration depth of the mirrors have been neglected. The former since the systems in this work use light in the communication band (around 1.55 µm), for which there are mirrors with a minimum of (linear) dispersion. The latter since the effect is smaller than the uncertainty of the molar polarizability of the gas [27,28].

### 2.2. Molar Density

For pressures below one atmosphere, the molar density can be calculated by assessing the refractive index and using the extended Lorentz–Lorenz equation

(3) $$\rho =\frac{2}{3A_{R}}\left(n-1\right)\left[1+b_{n-1}\left(n-1\right)\right],$$

where $A_{R}$ and $b_{n-1}$ are the molar dynamic polarizability [6,19]. The latter is given by $-(1+4B_{R}/A_{R}^{2})/6$, where, in turn, $B_{R}$ is the second refractivity virial coefficient in the Lorentz–Lorenz Equation [16,27,29].

### 2.3. Pressure

The molar density can then be used to assess the pressure as

(4) $$P=RT\rho [1+B_{\rho }\left(T\right)\rho ],$$

where *R* is the ideal gas constant, *T* is the temperature of the gas, and $B_{\rho }\left(T\right)$ is the second density virial coefficient.

For more detailed theoretical descriptions of the Lorentz–Lorenz equation and the equation of state, and for expressions valid for higher pressures, the reader is referred to the literature, e.g., [2,5,16,27,29,30,31,32].

### 2.4. Molecular Data

In this work, all assessments were performed on nitrogen. Table 1 provides information about the relevant gas constants for nitrogen, $A_{R}$, $b_{n-1}$, and $B_{\rho }$.

### 2.5. Set Pressure of the DWPG

In this work a DWPG was used to provide a pressure by loading a known mass on a piston-cylinder ensemble with a known area. The pressure was calculated as

(5) $$P_{DW}=\frac{\left(m_{p}+\sum_{i}m_{i}\right)g\cdot cos\left(\theta \right)}{A_{eff}\left[1+\alpha \left(T_{p}-T_{ref}\right)\right]}+P_{hood},$$

where $m_{p}$ is the mass of the piston, $m_{i}$ is the mass of the individual weights, *g* is the local gravity, θ is the angle between the piston cylinder assembly and the gravity vector, $A_{eff}$ is the effective area of the piston at the temperature $T_{ref}$, α is the combined temperature expansion of the piston and cylinder, $T_{p}$ is the measured temperature, and $P_{hood}$ is the hood pressure [33].

## 3. Experimental Setup

### 3.1. The Dual Refractometry System Used in This Work

**Figure 1:**
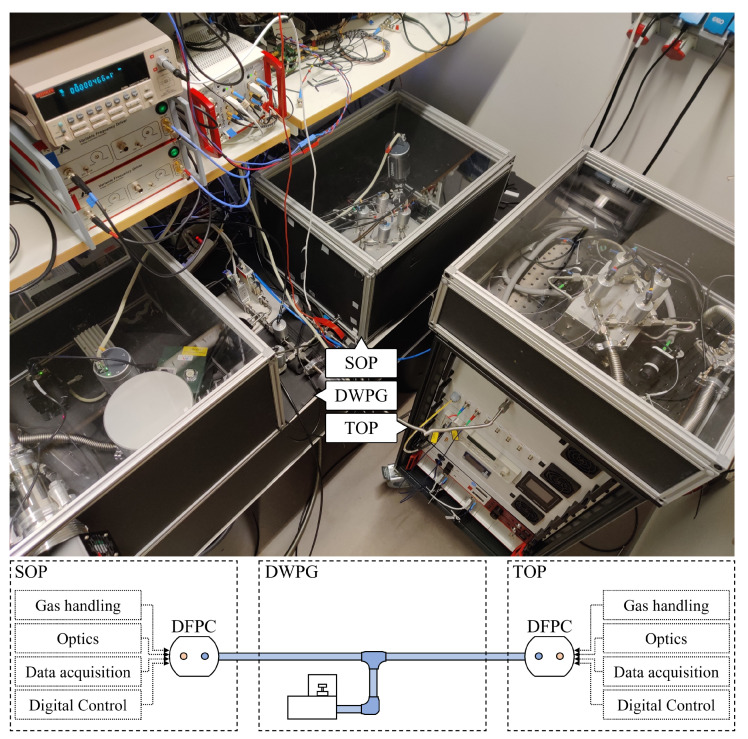
The dual refractometry system scrutinized in this work. It consists of three main components: the SOP (the system in the rightmost box on the optical table); the TOP-refractometer (the standalone system to the right); and the DWPG (the system in the leftmost box on the optical table). In addition to this, it comprises a common gas supply (seen between the SOP and DWPG boxes), a common vacuum system (not in the figure), a computer (for control and data acquisition), and various electronics—for the SOP, partly seen on the shelves, and for the TOP, in the rack. In the bottom part of the figure, a schematic showing the subsystems of the two refractometers and their connection to the DWPG is presented. The blue gas line represents the gas pressure under assessment and the red circles within the DFPCs represent evacuated cavities.

To demonstrate the short-term performance of GAMOR based refractometry, two fully independent Invar-based DFPC refractometers, the aforementioned SOP and TOP, were connected to a DWPG. Figure 1 shows a picture of the experimental setup. While the SOP was firmly placed on an optical table (placed in the rightmost box on the optical table, in the center of the figure), the TOP is designed to fit in a 19-inch transportable rack (the standalone unit to the right). They were both connected to a DWPG (placed in the leftmost box on the optical table).

The two refractometry systems are virtually identical in terms of optical and electronic components, including the FP-cavity ensemble. As shown in the schematics above, each system has its own gas handling system; optics (including lasers, electro-optics, passive optics, and locking electronics); and data acquisition and digital control in the form of digital to analogue converter (DAQ) modules and a computer. However, they differ when it comes to the means by which they assess the gas temperature: while the SOP system uses a thermocouple that refers to a miniature fixed point gallium cell to measure the spacer temperature [25], the TOP system assesses it through the use of calibrated Pt-100 sensors. Normally, when working individually, the two refractometry systems use their own designated gas supplies and vacuum systems. In this work, however, they were connected to a common gas supply and vacuum system.

### 3.2. The TOP Refractometer

**Figure 2:**
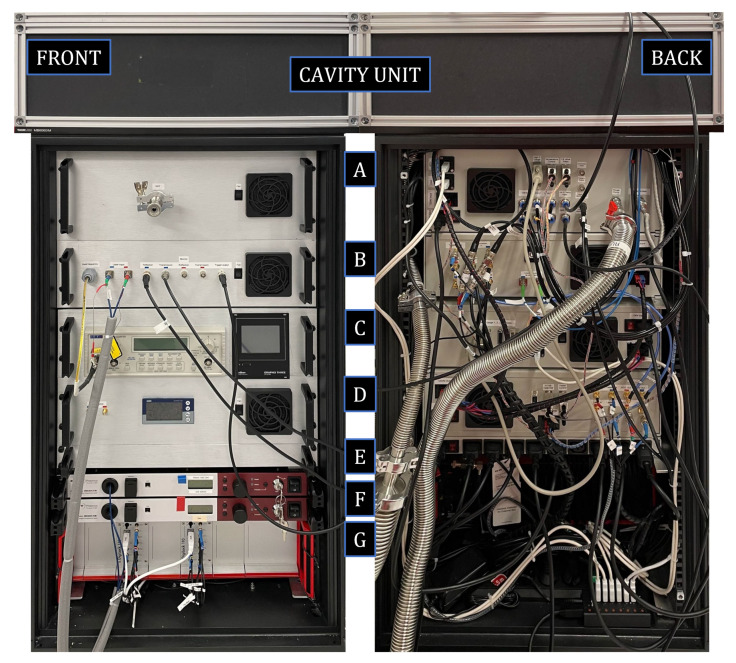
The TOP system seen from the front and rear. All lasers, electronics, and gas connections are placed within a 19-inch rack. On top of the rack, there is a 60 × 60 × 25 cm encapsulated box (denoted the cavity unit) that contains, as its base, an optical breadboard, on which the Invar-based DFPC is placed (in turn, encapsulated in an aluminum enclosure, denoted the "oven"). This unit also comprises four pneumatic valves that control the filling and emptying of gas in the cavity during the GAMOR-cycles (as can be seen in Figure 1, placed on top of the oven); and collimators, mirrors, and detectors that couple light into the cavities and measure the transmittance. The rack contains thereafter, from the top to the bottom, seven modules, denoted A–G, containing vacuum connectors, a communication hub, fiber-optics, a frequency counter, two fiber lasers, and locking electronics. The rack stands on four wheels that allow the system to be easily moved within the laboratory.

While the construction of the SOP, and the role of its various comprised parts, have previously been described in some detail in the literature [17,25], those of the TOP have not. Therefore, we describe those in more detail here. As is shown in Figure 2, the TOP refractometer system was built within a 19-inch transportable rack. For sturdy transportation and best serviceability, the system has been divided into a number of subsystems. From top to bottom, they comprise a top unit (denoted the cavity unit) and seven subrack enclosures, denoted the modules (A–G), comprising a number of separate mechanical, optical, and electrical entities that play the same role in the system as the corresponding parts do in the SOP system [17,25].

The cavity unit consists of a 60 × 60 cm breadboard that is firmly attached to the top of the rack. The DFPC Invar spacer sits at the center of this breadboard within an aluminum enclosure (oven) that is temperature controlled by four Peltier elements. The breadboard is, in turn, temperature regulated by a heat mat placed under it. Mounted to the breadboard, surrounding all components in this unit, there is a 60 × 60 × 25 cm aluminum framework with thermally isolated walls (shown in the figure).

Four pneumatic valves, used to control the flow of gas into and out of each cavity, are attached to the top of the oven. As is further described in Section 3.3, two of these valves (denoted $V_{T.1}$ and $V_{T.3}$) are connected to the gas supply unit, and two ($V_{T.2}$ and $V_{T.4}$) are connected to a turbo pump. To provide an assessment of the reference pressure, a pressure gauge (denoted $G_{T.2}$ in Section 3.3) is mounted on the turbo line (in close proximity to the reference cavity).

The cavity unit also contains customized fiber collimators that mode match the light from the lasers into the cavities; mirrors that direct the light; and detectors (Thorlabs, PDA50B-EC), placed behind each cavity, that detect the light transmitted on resonance.

Module A comprises the gas inlet system, consisting of a mass flow controller and an electronic pressure controller (denoted MFC and EPC in Section 3.3, respectively) that provide a continuous flushing of gas and regulation of the pressure; and a pressure gauge, $G_{T.1}$, that provides a rough assessment of the pressure under scrutiny. It also contains a four slot compact DAC (CompactDAQ, National Instruments, cDAQ-9174) that holds an analogue input module (National Instruments, NI-9215) to monitor the feedback voltages sent to the lasers; a temperature input module (National instruments, NI-9216) to measure the Pt-100 readings; a voltage output module (National Instruments, NI-9263) to give feed back to the Peltier drivers; and a digital output module (National Instruments, NI-9474) to control the pilot valves (which also resides in Module A). The front panel is equipped with a VCR port to connect the device to be scrutinized by the TOP (the device under test, DUT).

The rear panel is equipped with 230, 24, and 12 V power supply inputs (in the leftmost part of the figure). Above these, there are two USB connectors to the cDAQ and the MFC/EPCs. At the center there are eight push-in 6 mm pneumatic fittings to provide pressurized air to the seven pilot valves and the gas to the supply unit. Above these, there are three D-sub connectors, which are used to connect the high pressure gauge to the vacuum gauge controller (Oerlikon-Leybold, Graphix Three); the cDAQ with the Peltier driver; and a fill pressure relay with the gas filling valve. In the rightmost part of this panel there are two gas connectors: one VCR that is connected to the valve system inside the cavity unit at the top of the rack, and one Swagelok connector that can be used for rough pumping of the gas system.

Module B contains most of the optics, passive fiber optical components (e.g., circulators and isolators), and opto-electronics. The leftmost part of the front panel comprises the output from the beat detector and the input for the fibers from the lasers. The light that enters via the fibers is coupled into acousto optic modulators (AOM, AA Opto-Electronic, MT110-IR25-3FIO), after which it is coupled into 90/10 splitters. The light in the 10% outputs of the two splitters is coupled to the beat detector (Thorlabs, PDA8GS) via a 50/50 combiner. The light in the 90% outputs is coupled into electro-optic modulators (EOM, General Photonics, LPM-001-15) for the production of sidebands for the Pound–Drever–Hall locking. The light fields are then coupled into circulators via isolators (to prevent back reflections to the EOM). The forward output of each circulator is coupled via a fiber to the collimator for further passage into the cavity unit, and their rear outputs, which monitor the reflections from the cavities, are connected to reflection detectors (Thorlabs, PDA10CE-EC). The front panel is also equipped with five BNC-ports that are connected to the transmission and reflection detectors of the system. The fifth of these is used as a trigger that enables an oscilloscope to be connected to the other ports for the alignment procedure of the free space optics in the cavity unit.

On the rear panel, nine SMA-connectors are positioned to the left, comprising the control signals for the EOM and AOM; the inputs from the transmission detectors (for the monitoring port on the front panel); and the outputs from the reflection detectors (for the feedback signal to the automatic locking unit). The ninth port is the trigger input from a digital laser locking module (Toptica, DigiLock 110). At the center are the circulator outputs, which are connected to the collimators in the cavity unit, and a USB port (not connected).

Module C holds a frequency counter (Aim-TTi, TF960) that measures the beat frequency detected by the beat detector (positioned in module B) and the vacuum gauge controller that is used to monitor the pressure gauges within the system (the $G_{T.1}$ monitoring the pressure under scrutiny and $G_{T.2}$ recording the reference pressure). The frequency counter and gauge controller are digitally controlled and monitored but can also be reached manually, as their fronts are shown on the front panel of this module. The back panel comprises three D-sub connectors, which are used to connect the two pressure gauges and the fill pressure relay, and two USB-ports, which are the communication interface for the frequency counter and the vacuum gauge controller.

Module D comprises various 12 and 24 V power supplies; the custom made voltage controlled oscillators, which regulate the AOMs in module B; servo circuits for the locking of the lasers to the cavity modes; and the control unit for the heat mat (JUMO, diraTRON 108) that regulates the breadboard under the cavity unit, seen at the center of the front panel. The front panel is also equipped with two SMA ports for monitoring of the VCOs.

In the center of the back panel, the output for the driving current of the Peltier elements and the heat mat can be found. The rightmost part of the back panel comprises the inputs and outputs for the VCO and Laser PZT voltages.

Module E is a power distribution unit, in which the main 230 V input is split into nine 230 V outputs for power distribution to each subsystem.

Module F consists of two Er-doped fiber lasers (EDFL, NTK, Koheras Adjustik E15) that produce the light that is coupled, through fibers, to module C.

Module G, on the very bottom, contains the two digital laser locking modules (Toptica, DigiLock110) used for the automatic locking procedure of the lasers to their respective cavity.

Finally, at the bottom right on the back side of the rack there is a USB-hub that connects various electronics to a laptop accompanying the TOP system. The system is controlled by the laptop that, through the use of custom made LabVIEW software, gathers all data required for analysis.

### 3.3. The Gas Handling System for the Dual Refractometry System Used in This Work

**Figure 3:**
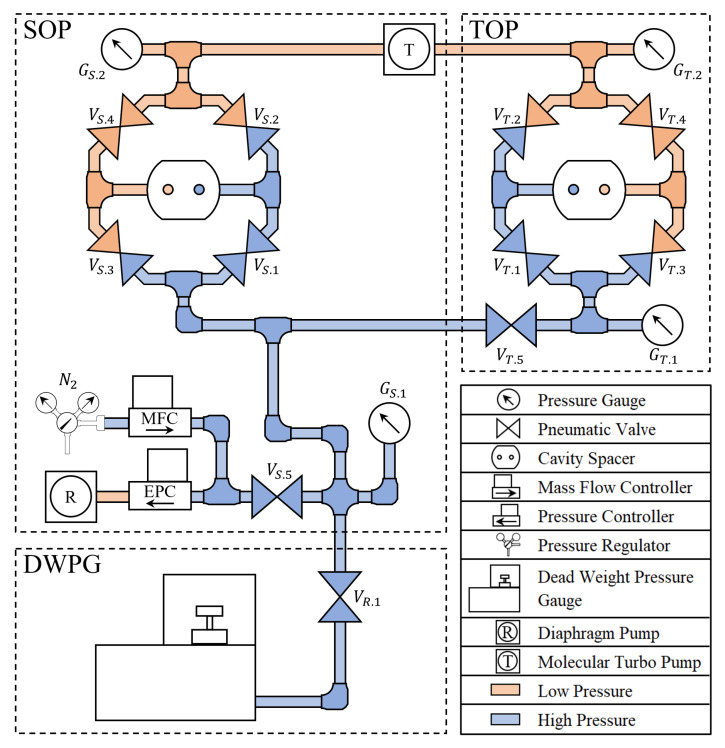
Schematic view of the gas delivery system. The components are described by the legend in the lower right corner. The setup is divided into three sub-systems, viz., the SOP, the TOP, and the DWPG. The SOP, presented in the upper left corner, regulates and controls the gas filling unit, consisting of the MFC and EPC. It also controls the primary fill valve, $V_{S,5}$. The valves $V_{S.1-4}$, which control the filling and evacuation of the cavities, are opened or closed in a given sequence. This subsystem also comprises two pressure gauges: $G_{S.1}$, which monitors the high pressure side; and $G_{S.2}$, which measures the residual pressure on the low pressure side. The TOP, which is displayed in the upper right corner, applies the same logic as the SOP system to its valves ($V_{T.1-4}$) and gauges ($G_{T.1-2}$). This subsystem can additionally be connected to or disconnected from the gas delivery system by use of valve $V_{T.5}$. Finally, the DWPG is presented in the lower left corner. This is connected or disconnected to the rest of the system by the valve $V_{R.1}$. In this presentation, the systems are displayed with the filling/connecting valves, $V_{S.5}$, $V_{S.1}$, $V_{T.5}$, $V_{T.1}$, and $V_{R.1}$ open. This implies that the measurement cavities are filled by the pressure set by the DWPG (represented by the blue gas lines). In addition, the valves $V_{S.4}$ and $V_{T.4}$ are open to allow for an evacuation of the reference cavities (represented by the red gas lines). The measurement cycle is followed by an evacuation state in which the valves $V_{S.1}$ and $V_{T.1}$ are closed and $V_{S.2}$ and $V_{T.2}$ are opened, which evacuates all cavities.

Figure 3 shows a schematic view of how the two refractometers are connected to the gas system comprising a common gas supply and distribution system.

In the figure, the colored lines represent gas tubes where the red color relates to low pressures while the blue represents high pressures. To fill the system with gas, the gas supply system, consisting of a mass flow controller (MFC, Bronkhorst, FG-201CV) and an electronic pressure controller (EPC Bronkhorst, P-702CV), is connected to a supply (in this work $N_{2}$). In the volume to the left of valve $V_{S,5}$, gas constantly circulates to prevent contamination build up.

When the refractometers are to be filled with gas, the valves $V_{S/T.5}$ and $V_{S/T.1}$ are opened. Valve $V_{S,5}$ is opened and closed by a relay controlled by switching the set-point of the vacuum gauge controller. The input for the set-point is the pressure measured by gauge $G_{S,1}$ (Oerlikon-Leybold, CTR 101 N 1000 Torr) in the SOP-refractometer and a set pressure chosen "close" to the nominal set value of the DWPG (as given by Equation (5)). This setup means that the gas system will be re-pressurized whenever the pressure drops below the chosen set pressure. After the re-pressurization the DWPG will automatically regulate the pressure to its set-pressure. During the gas filling and stabilization stage, the valves $V_{S/T.2}$ and $V_{S/T.3}$ are closed, and the valves $V_{S/T.4}$ are open, resulting in evacuation (close to vacuum) of the reference cavities in both of the refractometers, represented by the red gas lines. When both measurement cavities are to be evacuated, the valves $V_{S/T.1}$ are closed and the valves $V_{S/T.2}$ are opened, leading to the evacuation of all cavities.

The gas lines are not depicted at an appropriate scale; counted from the common tee (depicted above valve $V_{S,5}$) and the gas molecular turbo pump, the gas lines to the TOP are significantly longer than the corresponding ones to the SOP.

## 4. Methodology and Results

In order to perform the measurements presented in this work, the GAMOR methodology has been used. Although this methodology has previously been described in the literature [6,17,19], the following two sections, Section 4.1 and Section 4.2, give a brief overview of its principles. The latter one emphasizes how the methodology can be used to obtain pressure assessments in seconds. Section 4.3 provides a characterization of the two GAMOR-based DFPC refractometry systems used, and Section 4.4 gives an example of an assessment. Finally, Section 4.5 presents the results of a series of assessments.

### 4.1. Conventional Realization of the GAMOR Methodology

**Figure 4:**
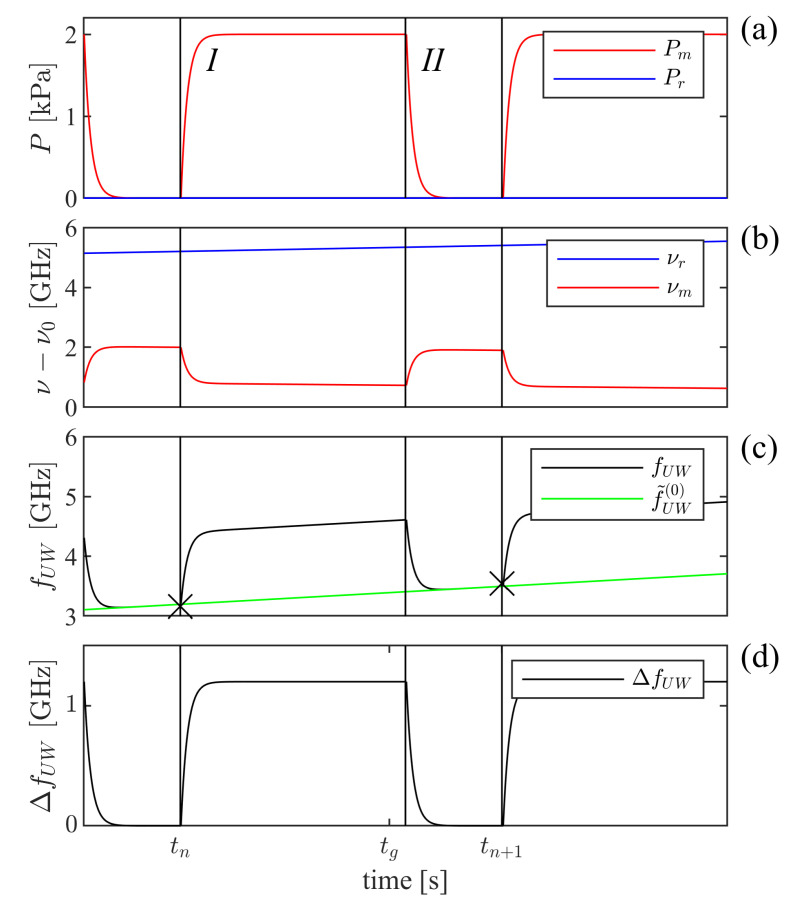
A schematic illustration of the principles of GAMOR implemented on a system exposed to campaign-persistent drifts. Panel (**a**) shows, as functions of time, by the the upper red curve, $P_{m}\left(t\right)$, the pressure in the measurement cavity, and by the lower blue curve, the pressure in the reference cavity, $P_{r}\left(t\right)$. Panel (**b**) depicts the corresponding frequencies of the measurement and reference lasers, $\nu_{m}\left(t\right)$ (the lower red curve) and $\nu_{r}\left(t\right)$ (the upper blue curve), respectively, for display purposes, both offset by a common frequency. Panel (**c**) displays, by the upper black curve, the corresponding unwrapped beat frequency in the presence of gas, $f_{UW}\left(t\right)$, and the lower green line depicts the estimated evacuated measurement cavity beat frequency, ${\tilde{f}}_{UW}^{\left(0\right)}\left(t_{n},t,t_{n+1}\right)$. Panel (**d**) displays the drift-corrected shift in unwrapped beat frequency, $\Delta f_{UW}\left(t\right)$. While the data that are used in ordinary GAMOR constitute the data points in the last part (10–20%) of section *I* (the time period between $t_{n}$ and slightly after $t_{g}$) in panel (**d**), which are averaged to a single data value, in this work where cycle resolved assessments are performed, a significant part (ca. 80%) of the data in section *I* is used in an unaveraged manner. Note that the drifts have been greatly exaggerated for clarity.

As has been indicated previously [6,19,34], the GAMOR methodology is based on two cornerstones: viz., (i) frequent referencing of filled measurement cavity beat frequencies to evacuated cavity beat frequencies, and (ii) an assessment of the evacuated measurement cavity beat frequency at the time of the assessment of the filled measurement cavity beat frequency by use of an interpolation between two evacuated measurement cavity beat frequency assessments, one performed before and one after the filled cavity assessments. The principles for the methodology when campaign-persistent drifts take place are schematically illustrated in Figure 4.

Figure 4a illustrates the pressure in the measurement cavity, which, according to cornerstone (i), is alternately evacuated and filled with gas (upper red curve), and the reference cavity is held at a constant pressure (lower blue curve). Campaign-persistent drifts will affect the frequencies of both the measurement and the reference lasers (although possibly to dissimilar extent, as shown in Figure 4b) and thereby both the assessed beat frequency, f(t), and its unwrapped counterpart, $f_{UW}\left(t\right)$ (the latter displayed by the upper black curve in Figure 4c). These curves indicate that the influence of drifts can be reduced by shortening the modulation cycle period; for a given drift rate, the shorter the gas modulation period, the less the assessed beat frequency will be affected by drifts [21].

Furthermore, according to cornerstone (ii), the unwrapped evacuated measurement cavity beat frequency is, for each modulation cycle, not assessed by a single measurement. It is instead estimated by the use of a linear interpolation between two evacuated (unwrapped) measurement cavity beat frequency assessments performed in rapid succession—one taken directly prior to when the measurement cavity is filled with gas (for cycle *n*, at a time $t_{n}$, denoted $f_{UW}^{\left(0\right)}\left(t_{n}\right)$), and another directly after it has been evacuated (at a time $t_{n+1}$, denoted $f_{UW}^{\left(0\right)}\left(t_{n+1}\right)$), both marked by crosses in Figure 4c. By this, the unwrapped evacuated measurement cavity beat frequency, ${\tilde{f}}_{UW}^{\left(0\right)}\left(t_{n},t,t_{n+1}\right)$, can be estimated at all times *t* during a modulation cycle. For cycle *n*, for which $t_{n}\leq t\leq t_{n+1}$, it is estimated as

(6) $${\tilde{f}}_{UW}^{\left(0\right)}\left(t_{n},t,t_{n+1}\right)=f_{UW}^{\left(0\right)}\left(t_{n}\right)+\frac{f_{UW}^{\left(0\right)}\left(t_{n+1}\right)-f_{UW}^{\left(0\right)}\left(t_{n}\right)}{t_{n+1}-t_{n}}\left(t-t_{n}\right).$$

For the case with campaign-persistent drifts, this interpolated value is represented by the green line in Figure 4c.

By subtracting the estimated (interpolated) unwrapped evacuated measurement cavity beat frequency (${\bar{f}}_{UW}^{\left(0\right)}\left(t_{n},t,t_{n+1}\right)$, the green line) from the measured (drift-influenced) unwrapped beat frequency during gas filling ($f_{UW}\left(t\right)$, the black curve), both in Figure 4c, a campaign-persistent, drift-corrected net beat frequency, represented by the black curve in Figure 4d, can be obtained. The average value of this curve a short time period just before the cavity is evacuated, at a time denoted $t_{g}$, represents the $\Delta f_{UW}$ to be used in the Equation (2) when GAMOR is performed. This shows that it is feasible to interpret GAMOR as "interpolated gas modulated refractometry."

### 4.2. Use of GAMOR to Assess Short-Term Pressure Fluctuations

GAMOR refractometry has so far been used to assess static pressures through the use of (and averaging over) a series of gas modulation cycles. It has been shown, for example, that, for the case with an Invar-based DFPC system, a minimum deviation could be achieved when averaging was performed over ten modulation cycles (i.e., over $10^{3}$ s) [17]. Such a mode of operation is suitable when static pressures (or slowly varying pressures, those that change slowly over time intervals corresponding to several gas modulation periods) are to be assessed. In such a case, the methodology first calculates a single pressure value for each individual gas modulation cycle (as schematically described in Figure 4) and then takes the average over *n* such cycles. For the case when the instrumentation is mainly affected by white noise, this process will improve on the precision (decrease the influence of noise) by a factor of $n^{-1/2}$.

The GAMOR methodology can though also be used for assessing short-term fluctuations of pressure. In this case, the assessment of the pressure, P(t), is continuously carried out from the shift in the incessantly assessed unwrapped beat frequency, $\Delta f_{UW}\left(t\right)$, during individual modulation cycles. A calculation of the cycle resolved pressure began by assessing, for each refractometer, from the beat frequency, f(t), and the shifts in cavity mode numbers $\Delta q_{1}\left(t\right)$ and $\Delta q_{2}\left(t\right)$, the unwrapped (i.e., the mode-jump-corrected) beat frequency, $f_{UW}\left(t\right)$. The beat frequency was continuously sampled by a frequency counter with a readout rate of 4 Hz. The shift in cavity mode number $\Delta q_{i}\left(t\right)$ was calculated as the nearest integer to $\left[\left(V_{i}\left(t\right)-V_{0,i}\right)/V_{FSR,i}+q_{0,i}\left(P_{cav,i}\left(t\right)/P_{0}\right)\left(n_{0}-1\right)\right]$, where $V_{i}\left(t\right)$ is the voltage sent to the tuning control of laser *i* (acting on its piezo stretcher), $V_{0,i}$ is the voltage for an empty cavity locked to mode $q_{0,i}$, $V_{FSR,i}$ is the voltage required to tune the laser FSR, $P_{cav,i}\left(t\right)$ is the pressure in the cavity *i*, and $n_{0}-1$ is the refractivity for the gas addressed that corresponds to the pressure $P_{0}$ (here taken as $10^{5}$ Pa). The pressure $P_{cav,i}\left(t\right)$ is assessed by the use of pressure gauge $G_{S/T.1}$. Hence, by continuously measuring the voltages sent to the lasers, all information needed to calculate the changes of the cavity mode numbers, $\Delta q_{1}\left(t\right)$ and $\Delta q_{2}\left(t\right)$, is available at all times. Using this information and Equation (1) it is possible to calculate the unwrapped beat frequency, $f_{UW}\left(t\right)$, at all times during a modulation cycle.

### 4.3. System Characterizations

Prior to the measurements, the two refractometers were first individually characterized by assessing the cavity deformations by the use of the methodology presented in [26]. The results are presented in detail in [19]. It was found that the cavity deformation parameters, $\varepsilon_{m}$, for the SOP and TOP when assessing nitrogen, were 0.001972(1) and 0.001927(1). Since (n−1)∝$\left(1-\varepsilon_{m}\right)$, the measurement uncertainty in the cavity deformations will solely contribute to the total expanded uncertainty in pressure (k = 2) with 1 ppm. Furthermore, using a thorough evaluation, the two refractometers were attributed expanded uncertainties (k = 2) for assessment of pressure of nitrogen, of ((10 mPa)${}^{2}$ + (10 × 10${}^{-6}{P)}^{2}$)${}^{1/2}$ for the SOP and ((16 mPa)${}^{2}$ + (28 × 10${}^{-6}P{{)}^{2})}^{1/2}$ for the TOP [19]. It was found that while the SOP is predominantly limited by the uncertainty in the molar polarizability of nitrogen (8 ppm), the accuracy of the TOP is limited by the uncertainty of the temperature probes used for the temperature assessment (26 ppm). It should be noticed though, that both refractometers had smaller evaluated uncertainties than that of the DWPG, which was assessed to be ((60 mPa)${}^{2}$ + (41 × 10${}^{-6}{P)}^{2}$)${}^{1/2}$.

### 4.4. Cycle Resolved Pressure Assessment

**Figure 5:**
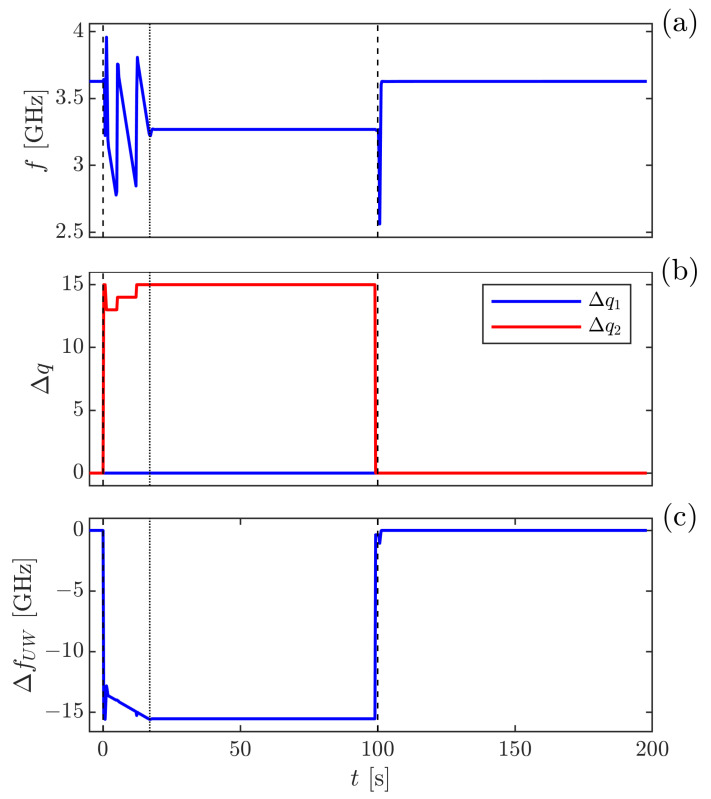
The time evolution of (**a**) the raw beat frequency [f(t)]; (**b**) the evaluated shift in mode number ($\Delta q_{1}\left(t\right)$ and $\Delta q_{2}\left(t\right)$); and (**c**) the corresponding unwrapped beat frequency ($f_{UW}\left(t\right)$) from the SOP over a 200 s long modulation cycle. For descriptions of the various time intervals of the modulation cycle, see the caption of Figure 6.

**Figure 6:**
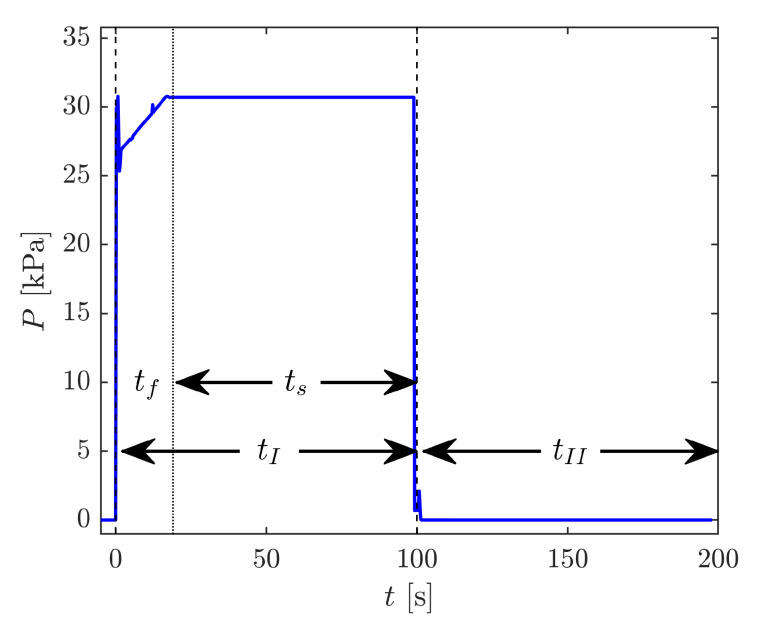
The time evolution of the pressure assessed in the SOP during the 200 s long gas modulation cycle displayed in Figure 5, P(t). $t_{I}$ represents the time of filling and $t_{II}$ the time of evacuation during the modulation cycle, here set to 100 and 100 s, respectively. $t_{f}$ is the time at which the MFC was re-filling the DWPG, and $t_{s}$ is the time during which the DWPG was stabilizing the pressure (i.e., when the piston was floating).

Figure 5 shows cycle resolved raw data from a single cycle from the SOP with a set pressure of the DWPG of 30.7 kPa; Figure 5a displays the measured beat frequency, f(t), Figure 5b shows the changes in cavity mode numbers, $\Delta q_{i}\left(t\right)$, and Figure 5c illustrates the calculated shift of the unwrapped beat frequency $\Delta f_{UW}\left(t\right)$. This shows that although mode jumps are seen as steps in the beat frequency in Figure 5a, when the measured shifts in cavity mode numbers displayed in Figure 5b is taken into account, the unwrapped beat frequency illustrated in Figure 5c is a continuous function. The gas modulation had a cycle time of 200 s, distributed over a filled and an evacuated measurement cavity cycle, both lasting 100 s (denoted $t_{I}$ and $t_{II}$ in Figure 6, respectively).

The filled measurement cavity cycle was initiated at 0 s by the closing of valve $V_{S.2}$ and an opening of valve $V_{S.1}$, which results in a fast increase of the pressure. The MFC was then filling the system (resulting in a constant increase of the pressure) for a time of 20 s (referred to as $t_{f}$ in Figure 6), until the set pressure was reached.

After the set pressure was reached, the piston in the DWPG was floating, which resulted in a stabilization of the pressure at a constant pressure for 80 s (denoted $t_{s}$ in Figure 6, given by $t_{I}-t_{f}$). The filled measurement cavity assessment, $f_{UW}\left(n,t\right)$, was measured during the last 20 s of this period.

Thereafter, valve 1 was closed and valve 2 was opened, which resulted in a fast decrease in pressure (an increase in the unwrapped beat frequency). Both cavities were then evacuated for 100 s. The empty measurement cavity assessment, $f_{UW}\left(n=1,t\right)$, was measured during the last 20 s of this period. After this, the cycle was repeated.

These signals were then converted into pressure by Equations (2)–(4) and (6). Figure 6 shows the cycle resolved pressure calculated by these means from the $f_{UW}\left(t\right)$ data displayed in Figure 5. Note that the data in Figure 6 include several mode jumps during the filling ($t_{f}$) and the emptying stages that produced short "sparks" in the unwrapped pressure. Since the evaluation procedure did not use data points during the these stages (i.e., when the mode hops take place), they did not affect the final assessments.

### 4.5. Evaluation of the Degree of Short-Term Concordance between the Two Refractometers

To properly evaluate the degree of short-term concordance between assessments made by the two refractometers, they were jointly connected to the DWPG while extraordinarily long gas modulation cycles (300 s) were used (each comprising filling and evacuation periods of 250 and 50 s, respectively). A series of 20 such gas modulation cycles (which thus took 100 min) were performed. To reduce the load on the turbo pump, which was significantly affected by the repeated out pumping of gas at high pressure, this evaluation was performed at a set pressure of 16 kPa.

**Figure 7:**
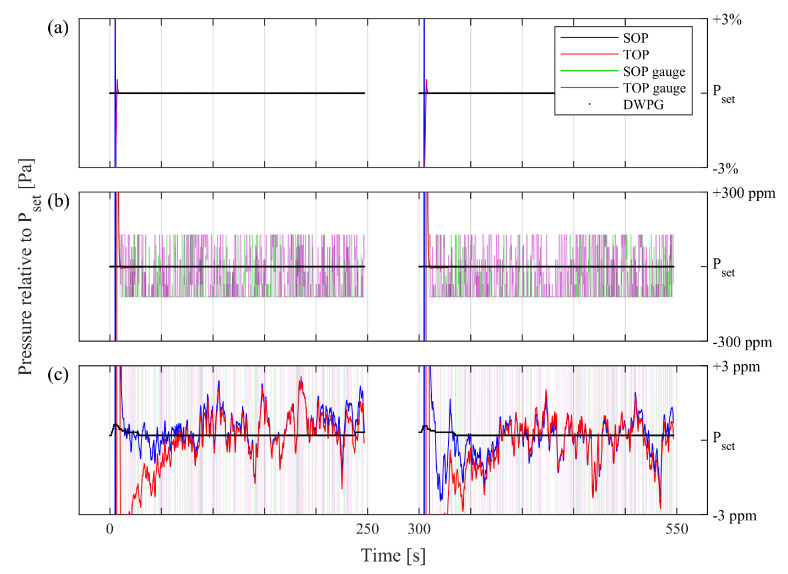
The 250 s parts of two consecutive (300 s long) gas modulation cycles when the measurement cavities contain gas for a DWPG set pressure of 16 kPa. The three panels display the same data centered around the set pressure but with dissimilar y-scales: in panel (**a**) with a scale of ±3%; panel (**b**) with a scale of ±300 ppm; and panel (**c**) with a scale of ±3 ppm. Black curves: the DWPG; green curves: the SOP gauge; purple curves: the TOP gauge; blue curves: the SOP; and red curves: the TOP.

Figure 7 shows two typical consecutive modulation cycles. Figure 7a–c encompass the same information, although Figure 7b,c are zooms of the data with 10${}^{2}$ and 10${}^{4}$ times magnification of (a), respectively.

The set pressure of the DWPG, estimated by use of Equation (5), is marked with the black (almost fully horizontal) curve. The pressure readings from the pressure gauges in the SOP and TOP systems, $G_{S.1}$ and $G_{T.1}$, respectively, are represented by the green and purple curves, respectively. The SOP and TOP refractometry signals are represented by the blue and red curves, respectively. In this comparison, the gauges and the refractometers have, for clarity, been adjusted by an offset (for SOP and TOP by 0.11 and 0.12 Pa, respectively) to overlap the set pressure of the DWPG. It is worth noting that said adjustments are well within their uncertainty budgets and the uncertainty of the DWPG (from the uncertainty presented in Section 4.2, at 16 kPa the SOP, TOP, and DWPG uncertainties are 0.16, 0.45, and 0.66 Pa, respectively). It also does not affect their short-term performance.

It is noteworthy that, in Figure 7a,b, the refractometer signals are not visible. This is due to the fact that they fluctuated less than the thickness of the DWPG curve and are hence hidden behind it. In Figure 7b, the pressure gauge readings (the green and purple curves) show bit noise; i.e., they fluctuate between two bits, an amount of 4 Pa (corresponding to 250 ppm of 16 kPa).

In Figure 7c, the gauges are off-scale, but the fluctuations of the refractometer signals are clearly visible. It is also worth noting that although there are significant fluctuations in both refractometer signals, there is a large degree of concordance between them. There is a slight tendency (predominantly seen during the first 50 s of the cycles) that the response of the TOP drifts with respect to that of the SOP. This is attributed to the fact that the gas lines to the TOP, because of practical reasons, had to be significantly longer than those to the SOP. This implies that the evacuation of the TOP during the evacuation cycle was not as efficient as that of the SOP. Hence, when the gas filling cycle commenced, there was a slightly higher residual pressure in the reference cavity of the TOP than that of the SOP. During the gas filling cycle, in which the data in Figure 7c were taken, and during which the reference cavities were constantly evacuated, the reference cavity of the TOP was further pumped down, resulting in an artificial drift of the TOP signal during the first 50 s of the gas filling cycle. It is important to note that this neither affects the level of correlation, nor does it imply that the GAMOR refractometers drift under normal working conditions; this drift takes place only because of the fact that the TOP system in this work, because of practical reasons, had to be connected to the gas system with unusually long gas lines.

**Figure 8:**
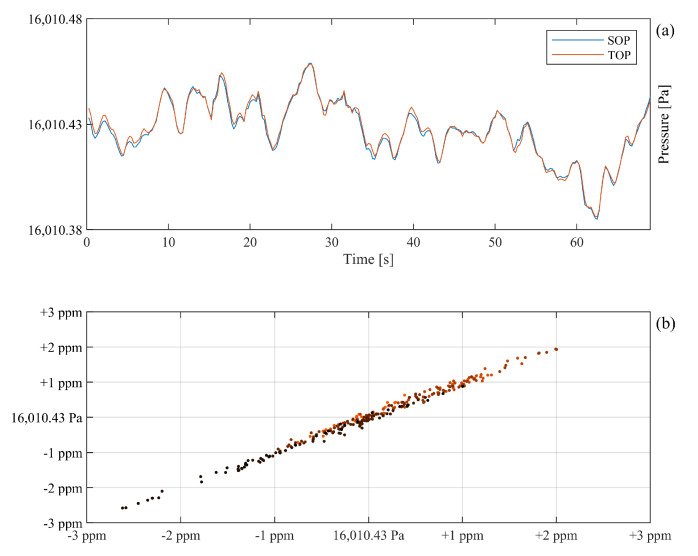
(**a**) An enlargement of 70 s of the refractometry data shown in the first cycle of Figure 7. (**b**) A correlation plot of the same data. The *x* and the *y*-axes represent the pressures assessed by the SOP and the TOP, respectively. In the latter, time is represented by the color, where the first data points are marked with orange and the last ones are in black.

To emphasize the degree of concordance of the two refractometer signals, 70 s of the pressures assessed by the refractometers depicted in the first cycle of Figure 7 are plotted at an enlarged scale in Figure 8a.

The degree of concordance between the two refractometer signals in Figure 8a is striking and impressive; they jointly and concurrently illustrate a common fluctuating pressure. The fact that they detected in unison the same fluctuation indicates that the signals originated from the DWPG and gas delivery system rather than from the refractometers.

To assess the degree of correlation between the two refractometer signals, they are plotted against one another in Figure 8b. The data show that while the pressure produced by the DWPG and the gas delivery system fluctuated more than 5 ppm, the two refractometer signals differed significantly less than 0.5 ppm. A correlation analysis of the data provides a remarkable correlation coefficient of the two refractometry signals of 0.995.

**Figure 9:**
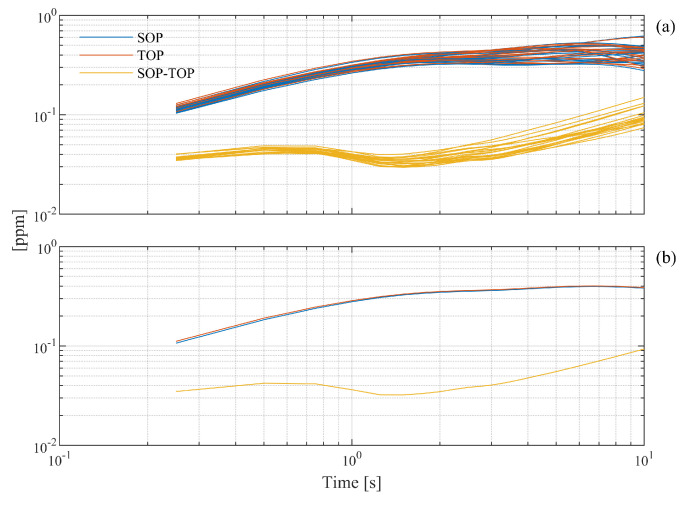
(**a**) The Allan deviation (as a function of averaging time) for the SOT refractometer (blue curves), the TOP refractometer(red curves), and their difference (yellow curves) assessed over 20 cycles. (**b**) The average of each set of data in panel (**a**).

To complement the correlation analysis above, the measurement data were also exposed to an Allan variance evaluation. Figure 9 displays, in terms of Allan deviations, the pressure assessments made by the SOP (blue curves), the TOP (the red curves), and their difference (yellow curves). Figure 9a displays all 20 assessments separately, and Figure 9b shows their average.

This figure shows a large degree of concordance also between the Allan plots of the 20 individual assessments for both the SOP and the TOP (as well their difference). This indicates that both refractometry systems were stable over the total period of the measurements. The standard deviation of the individual readings of each refractometer (the left-most points in Figure 9b) was 0.1 ppm, and their difference only had a standard deviation of 0.04 ppm.

## 5. Conclusions and Discussion

This paper provided scrutiny of the short-term performance capabilities of refractometry instrumentation based on the GAMOR methodology. We did so by comparing two independent refractometry systems coupled to a common dead weight piston gauge (DWPG). In contrast to conventional GAMOR-based refractometry, in which static pressures are assessed by the use of (and averaging over) a series of gas modulation cycles, the short-term assessments are performed *within* individual modulation cycles. More precisely, the short-term response is scrutinized through the use of a methodology in which the pressure, P(t), is continuously assessed by the shift in the incessantly measured unwrapped beat frequency, $\Delta f_{UW}\left(t\right)$, during individual modulation cycles. By this, GAMOR based instrumentation can assess fluctuations of pressures on time scales below the gas modulation time period. In addition, the methodology allows for an investigation of the ability of GAMOR to assess short-term fluctuations of pressures without any influence from the pressure producing and gas delivery system.

Figure 5 and Figure 6 display the typical cycle resolved response from one of the GAMOR instrumentations. The data show that, despite discrete mode jumps, the unwrapped beat frequency provides a continuous signal. The data also show that, in agreement with findings to be mediated by Rubin et al. [22], the response settled, within the resolution of the figure, within a fraction of the gas modulation period (the data do not show any visible drifts over the 80 s long time period denoted $t_{s}$). This vouches for the possibility to assess rapid changes in pressure during this time period.

Figure 7 shows that the simultaneous assessments performed by the two refractometry systems had a high degree of correlation; the deviations in their assessments were not only significantly smaller than those provided by the pressure gauges (which are limited by bit noise on a level orders of magnitude above that of the refractometers), they were also markedly smaller than those of the pressure assessed by either of them. A similarly excellent correlation between the pressure assessments performed by the two refractometers is shown in Figure 8. This indicates that their precision is significantly better than the stability of the pressure they assess, which implies that the deviations of the assessed pressure are attributable to fluctuations in the pressure in the DWPG and the gas delivery system, rather than to the performance of the refractometers.

These fluctuations can potentially have several causes, e.g., the ambient pressure, the gas temperature, vibrations, or fluctuations in the pressure produced by the DWPG. However, since Figure 8 shows that they take place over seconds, most of these potential causes are improbable. Instead, we presently attribute the most likely cause of the pressure fluctuations to the pressure produced by the DWPG.

Since Figure 9b indicates that the deviation of the difference assessment for time scales up to a few seconds was 0.04 ppm, it can be concluded that the short-term deviation of the pressure assessment by the use of a single refractometer was 0.03 ppm. Although pressure assessments of 4303 Pa have been demonstrated with an Allan deviation of 0.08 ppm assessed over $10{}^{4}$ s (corresponding to a standard deviation) [17], this shows that the refractometer systems have better precision than what so far has been demonstrated.

Since data were collected at 4 Hz (given by the finite updating time of the frequency counter) and there was no averaging process in the data acquisition, the bandwidth of the assessments shown in Figure 7 was 2 Hz (given by the Nyqvist theorem). Fundamentally though, this was limited by the cavity linewidth which, in this work, was in the order of 10’s of kHz.

With the extraordinary temporal response of the refractometer, this type of instrumentation can not only be used to measure rapid pressure changes and fluctuations, to investigate processes giving rise to such, and resolve the difference between sensor responses and actual pressure changes, it can also be used for characterization of the dynamic responses of pressure gauges (such as Pirani gauges).

Another application is that if the pressure can be kept constant, e.g., within a system regulated by a DWPG or another type of pressure regulator, it can serve as an instrument to characterize the temporal responses of temperature sensors. Finally, under stable conditions, one can isolate acoustic effects in the infrasound region, and hence be used in relation to the dB-scale.

## Figures and Tables

**Table 1:** Gas coefficients for N${}_{2}$ at 302.91 K and 1550.14 nm.

Coefficients	Value (k = 2)	Reference
$A_{R}$	4.396549(34) × 10${}^{-6}$ m${}^{3}$/mol	[26,32]
$b_{n-1}$	−0.195(7)	[15,26]
$B_{\rho }$	−4.00(24) × 10${}^{-6}$ m${}^{3}$/mol	[26,32]

## Data Availability

Data is contained within the article.
